# The economic impact of epilepsy: a systematic review

**DOI:** 10.1186/s12883-015-0494-y

**Published:** 2015-11-25

**Authors:** Katharina Allers, Beverley M. Essue, Maree L. Hackett, Janani Muhunthan, Craig S. Anderson, Kristen Pickles, Franziska Scheibe, Stephen Jan

**Affiliations:** The George Institute for Global Health, University of Sydney, Level 10, King George V Building, 83-117 Missenden Rd, PO Box M201, Camperdown, NSW 2050 Australia; University of Bremen, Bibliothekstraße 1, 28359 Bremen, Germany; The Menzies Centre for Health Policy, University of Sydney, D02 Victor Coppleson Building, Sydney, NSW 2006 Australia; Royal Prince Alfred Hospital, Level 11, KGV Building, Missenden Road, Camperdown, NSW 2050 Australia

**Keywords:** Epilepsy, Direct costs, Indirect costs

## Abstract

**Background:**

In this review we aimed to determine the economic impact of epilepsy and factors associated with costs to individuals and health systems.

**Methods:**

A narrative systematic review of incidence and case series studies with prospective consecutive patient recruitment and economic outcomes published before July 2014 were retrieved from Medline, Embase and PsycInfo.

**Results:**

Of 322 studies reviewed, 22 studies met the inclusion criteria and 14 were from high income country settings. The total costs associated with epilepsy varied significantly in relation to the duration and severity of the condition, response to treatment, and health care setting. Where assessed, ‘out of pocket’ costs and productivity losses were found to create substantial burden on households which may be offset by health insurance. However, populations covered ostensibly for the upfront costs of care can still bear a significant economic burden.

**Conclusions:**

Epilepsy poses a substantial economic burden for health systems and individuals and their families. There is uncertainty over the degree to which private health insurance or social health insurance coverage provides adequate protection from the costs of epilepsy. Future research is required to examine the role of different models of care and insurance programs in protecting against economic hardship for this condition, particularly in low and middle income settings.

**Electronic supplementary material:**

The online version of this article (doi:10.1186/s12883-015-0494-y) contains supplementary material, which is available to authorized users.

## Background

Epilepsy is one of the most common neurological conditions which occurs in about 5 to 8 cases per 1000 people per annum in developed countries [1, 2]. Epilepsy affects people of all ages and although treatable, often requires lifelong medication and sometimes surgery to control seizures [3]. The high health care costs related to assessment and treatment, surgery and hospitalisation for seizures, as well as lost employment, income, and household work, are well recognised [4]. These costs vary according to the severity of the condition, response to treatment, length of time since diagnosis, and the perspective examined (e.g. health systems, societal or individuals and families). However, the economic impact of epilepsy has been poorly quantified and few studies have evaluated strategies to reduce it [5].

A previous review of the economic impact of epilepsy in high and low and middle income countries was undertaken in 2008 and does not incorporate more recent studies [6] Most other reviews were limited to high-income settings and show associations with the temporal stage and severity of the disease, seizure frequency, drug treatment or resistance, hospital admissions and level of disability [7–11]. However, heterogeneous methods are used and study samples are small, raising issues of generalisability in particular towards those in low resource settings where rates of epilepsy are high [1]. Furthermore, the focus of previous reviews has largely been limited to the expenditure from the perspective of the health sector, excluding individual and household impacts. The aim of our review was to provide an update of the evidence and to examine the costs of epilepsy from societal, health system and household perspectives.

## Methods

Data were identified from published research articles and abstracts using a manual search in MEDLINE, Embase and PsycINFO databases, searched from inception to July 2014. We included incidence studies and case series with prospective consecutive patient recruitment within clearly defined geographical and time-limited boundaries. There were no restrictions on the basis of language, sample size, or duration of follow-up. Where articles were published in a language other than English, assistance was sought in translating the articles. Studies excluded were those limited to specific patient characteristics such as sex, where the recruitment strategy used convenience sampling with retrospective recruitment, were limited to unstructured assessment of psychosocial outcomes and focussed solely on clinical outcomes. If several articles reported outcomes from the same study population, data were taken from the first publication that referred to each follow-up period.

Two authors (KP, a public health researcher and Associate Professor MH, Head, Mental Health and Chronic Disease Program, Neurological & Mental Health Division) developed the search strategy using relevant terms that included common keywords for ‘epilepsy’ (e.g. epilepsy, epileptic, seizures, convulsions) combined with common keywords for ‘costs’ (e.g. economics, income, health care costs, expenses) (See Appendix for the search strategy). Epilepsy was defined as two or more recurrent unprovoked seizures. An epileptic seizure was diagnosed using the International League Against Epilepsy (ILAE) Commission on Epidemiology and Prognosis definition: “a transient occurrence of signs or symptoms due to abnormal excessive or synchronous neuronal activity in the brain.” [12] We also accepted any criteria the study authors used for epilepsy, including idiopathic epilepsy and epileptic syndromes with seizures of localized onset; symptomatic epilepsy and epileptic syndromes with simple partial seizures; symptomatic epilepsy and epileptic syndromes with complex partial seizures; and generalized idiopathic epilepsy and epileptic syndromes. Provoked, unprovoked, cryptogenic, remote, status epilepticus, febrile, convulsive, absence were also included.

The outcomes included were the costs to the health system (drugs, hospitalisations, visits to family doctor, etc.), costs to individuals and households (out- of-pocket costs and patient co-payments associated with treatment), and indirect costs in terms of lost income and production. The latter could be measured either in monetary units or some other measure such as time off work.

KP, MH and JM identified and reviewed papers for inclusion based on title and abstract in line with the inclusion criteria. The reference lists of all full text articles were reviewed to identify further articles of relevance that required retrieval of the full text. For all included studies (published full texts), a data extraction form was used to collect information on study design, setting, and outcome measures (Additional file 1) and data were extracted by one author (KA) and verified for accuracy (BE and SJ). The risk of bias in each study was assessed independently by the authors (KA, FS, MH and JM) using criteria based on a standard quality and risk-of-bias assessment [13].

Quantitative analysis was deemed inappropriate due to heterogeneity in the data, study designs and study settings. As this was a systematic review that did not involve data collection from participants, ethics approval was not required for this study.All information has been reported in accordance with PRISMA (Additional file 2) and MOOSE (Additional file 3) guidelines.

## Results

**Fig. 1:**
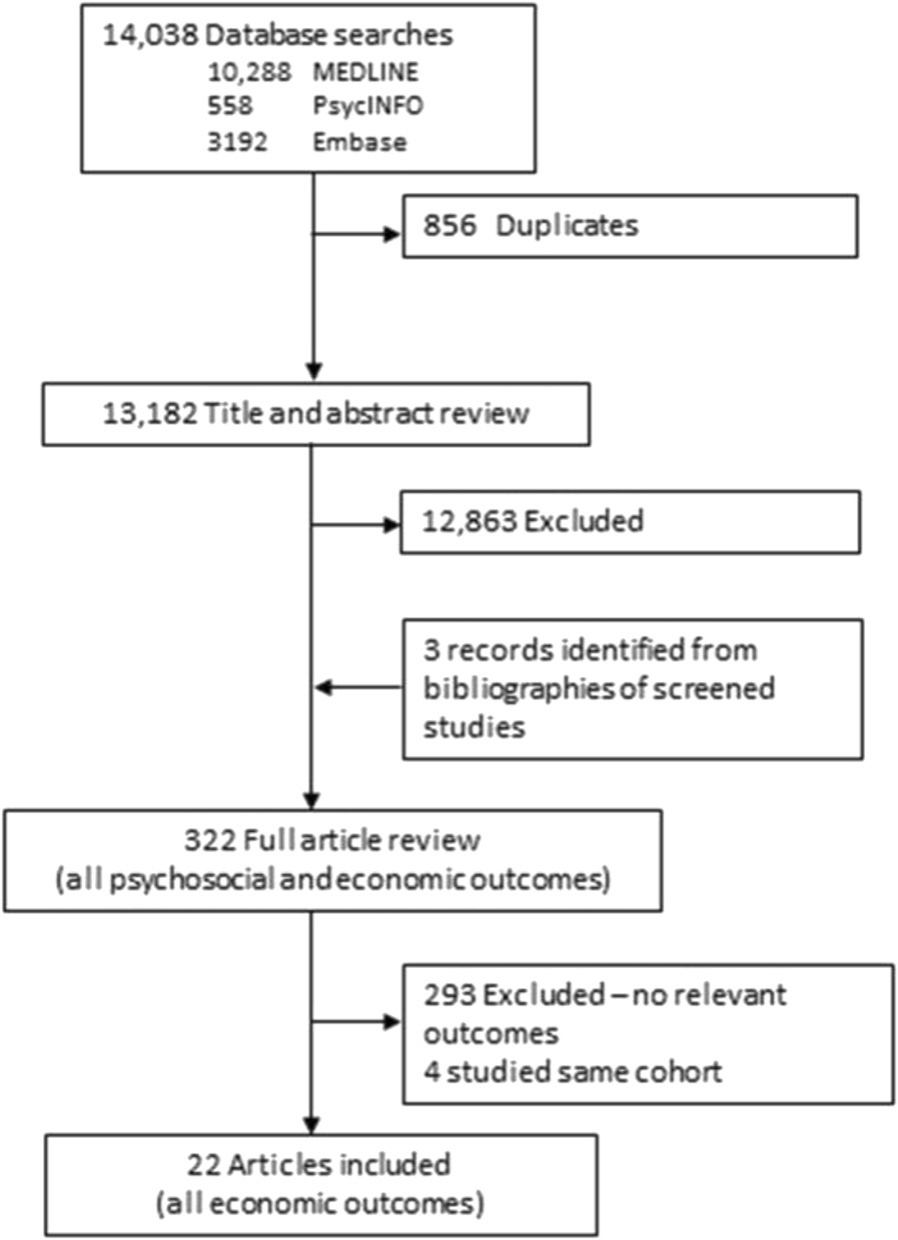
PRISMA Flowchart of included and excluded studies

Of 13588 papers retrieved, 322 studies were reviewed and 22 studies met the criteria for inclusion (see the PRISMA flowchart in Fig. 1). Table 1 summarises the characteristics of these studies where six were conducted from a health system perspective [14–19], seven were conducted from a societal perspective [4, 20–24, 33], and nine from an individual perspective [25–32, 34]. Seven studies factored in out-of-pocket costs incurred by patients [17, 29–34] and three studies reported productivity losses due to reduced work capacity [25, 26, 33]. Seventeen studies reported direct costs related to epilepsy (Table 2) and 10 studies estimated indirect costs (Table 3). The studies were conducted in 16 different countries; seven (from India, and Ecuador, Bulgaria, Nigeria, China, Cameroon and Cote d’Ivoire) were from low or middle income countries.

**Table 1:** Characteristics of studies included in review

Study	Study design	N	Country Patient population	Study objective	Follow-up period	Economic outcomes measured
Beghi et al. 2004 [14]	Cohort study	631	Italy 18 years of age and older NDE, SR, OS, NDR, DR or SC* (HIC)	To investigate the costs of epilepsy in different prognostic categories	12 months	- Direct costs
Boon et al. 2002 [15]	Cohort study	84	Belgium All ages Pre-surgical candidates who underwent a complete pre-surgical evaluation at Ghent University Hospital (HIC)	To compare and economically evaluate epilepsy-related direct medical costs incurred by different treatment modalities (conservatively, surgically and vague nerve stimulation- treated) and to determine.	Mean follow-up interval of 26 months	- Direct costs
Cockerell 1994 [4]	Cohort study	602	UK Newly diagnosed seizure disorder (sample of the National General Practice Study of Epilepsy (NGPSE) (HIC)	To assess the epilepsy related socio-economic costs in a population so that health care priorities can be set	Mean follow-up interval 6.6 years	- Direct costs
Das et al. 2007 [25]	Cohort study	1450	India No age indicated New patients with epilepsy in the Burdwan district (LMIC)	To evaluate the rate of discontinuation of epilepsy treatment and the related socio-economic factors responsible for discontinuation	12 months	- Direct costs - Indirect costs - Income
De Zelicourt et al. 2000 [20]	Cohort study	1942	France More than 1 month of age, Newly diagnosed unprovoked seizure (HIC)	Estimation of the direct medical cost for patients during the first two years after diagnosis	24 months	- Direct costs
Farmer et al. 1992 [26]	Quasi randomized trial	215	Ecuador No age indicated Epilepsy (identified in an epidemiological survey) (UMI)	To report the effects of epilepsy and its treatment on the social functioning of patients treated in Northern Ecuador	12 months	- Employment status
Guerrini et al. 2001 [16]	Cohort study	189	Italy Children and adolescents Followed up by child neurologist (university department, general hospital, outpatient department) (HIC)	To compare the direct costs of epilepsy in a child neurology referral population, stratified by disease, duration, and severity, across three health care settings.	12 months	- Direct costs of epilepsy
Halpern et al. 2011 [34]	Cohort study	574	USA All ages Epilepsy (HIC)	To assess whether people with epilepsy who are uninsured and those who have Medicaid coverage have greater out-of-pocket costs	6 years	- Out-of-pocket costs
Helmstaedter et al. 2000 [33]	Cohort study	161	Germany Adults Surgically or non-surgically treated patients with drug-resistant temporal lobe epilepsy (HIC)	To investigate the long-term effects of surgical and non-surgical treatment of drug-resistant temporal lobe epilepsy according to socioeconomic development	Mean follow-up interval 58 months	- Employment status
Kotsopoulos et al. 2003 [17]	Cohort study	116	Netherlands Age not indicated Established epilepsy, recruited from three patient populations (general practices, university hospital and epilepsy centre) (HIC)	(a) To gain insight into the direct and indirect costs of epilepsy care, and (b) To analyse the distribution of these costs by type of services for each patient group	3 months (and 3 months retrospective)	- Direct costs - Indirect costs - Out-of-pocket costs
Langfitt et al. 2007 [18]	Cohort study	68	USA. Age not indicated Temporal lobe epilepsy patients (HIC)	To determine whether health care costs change when seizures are controlled after surgery	2 years (and 2 years pre-evaluation)	- Direct costs
Lindsten et al. 2002 [21]	Case–control study	63	Sweden 17 years of age or older Newly diagnosed unprovoked seizure in Vaesterbotten, northern Sweden (HIC)	To investigate the socioeconomic prognosis after a newly diagnosed unprovoked epileptic seizure	10 years	- Income - Source of income - Sickness periods - Incapacity rate - Vocational status - Education
Pato Pato et al. 2011[22]	Cohort study	171	Spain Over 14 years of age Epilepsy (HIC)	To carry out an economic estimate of the direct, indirect and intangible costs of epilepsy	6 months	- Direct costs - Indirect costs - Intangible costs
Tetto et al. 2002 [19]	Cohort study	525	Italy All ages NDE, SR, OS, NDR, DR and SC from 14 epilepsy centres (HIC)	To compare the direct costs of epilepsy in patients referred with epilepsy of different severity and duration	12 months	- Direct costs
Balabanov et al. 2007 [24]	Cohort study	146	Bulgaria 18 years of age and older recruited from an epilepsy centre Epilepsy (UMIC)	To evaluate the effect of demographic and clinical factors on the quality of life and cost of treatment of epilepsy patients on monotherapy with carbamazepine and valproate	12 months	- Direct costs - Indirect costs
Lagunju et al. 2011 [32]	Cohort study	215	Nigeria Children over 18 months recruited from a paediatric neurology clinic Epilepsy (LMIC)	To estimate the total cost of childhood epilepsy and to provide essential information on the economic burden of childhood epilepsy in Nigeria	12 months	- Direct costs - Out-of-pocket costs - Indirect costs
Doumbia-Outtara et al. 2010 [31]	Cohort study	70	Cote d’Ivoire Adults recruited from an inpatient unit within a hospital department of neurology (LMIC)	To evaluate the efficacy and tolerance of anti-epileptic drugs and the financial cost of care	n/a	- Direct costs - Out-of-pocket costs - Indirect costs
Dongmo et al. 2003 [30]	Cohort study	125	Cameroon All ages recruited from a medical centre Epilepsy (LMIC)	To evaluate the difficulties faced in the management of epileptic patients in their natural environment	12 months	- Direct costs - Out-of-pocket costs
Haroon et al. 2012 [29]	Cohort study	134	India All ages recruited from a centre of neuroscience within a national hospital Epilepsy (LMIC)	To evaluate the costs of active epilepsy and study the pattern of drug prescription and utilisation in epileptic patients	4 months	- Direct costs - Out-of-pocket costs
Strzelcyck et al. 2013 [23]	Cohort study	252	Germany All ages recruited from an outpatient clinic within a university hospital Focal epilepsy (HIC)	To estimate the direct and indirect costs of epilepsy and evaluate trends in the resource use of patients with active epilepsy.	12 months	-Direct costs -Indirect costs
Lv et al. 2007 [28]	Cohort study	533	China Parents of children with epilepsy recruited from the outpatient clinic of a tertiary hospital epilepsy centre Epilepsy (UMIC)	To assess the impact of childhood epilepsy on parental quality of life (QoL) and psychological health, and to investigate possible correlations between parental QoL, background variables and parental anxiety and depression	12 months	- Direct costs - Income - Employment status
Vlasov et al. 2010 [27]	Cohort study		Russia Epilepsy (HIC)	To evaluate the clinical-economic effectiveness of anti-epileptic drug (AED) therapy	12 months	-Direct costs

*Abbreviations*: *DR* drug-resistant seizures, *NDE* newly diagnosed epilepsy, *NDR* frequent non-drug-resistant seizures, *OS* occasional seizures, *SR* seizure remission, *SC* surgical

World Bank country classifications: HIC: high-income country; UMIC: upper-middle income country; LMIC: lower-middle-income country

(Accessible at: http://data.worldbank.org/about/country-and-lending-groups#Low_income)

**Table 2:** Summary of findings – direct costs

Study	Total direct costs	Out-of-pocket costs	Direct cost summary
Beghi et al. 2004 [14]	Mean costs: €1302 Subgroups: NDE: €975; SR: €561 OS: €830; NDR: €1498; DR: €2568 SC: €3619	n/a	Costs of epilepsy patients vary significantly according to time course of the disease and response to treatment. Hospital admissions and drugs are major sources of expenditure
Boon et al. 2002 [15]	Conservatively treated Before: $ 2,525 After: $ 2,421 Surgically treated Before: $ 1,465 After: $ 1,186 Vagus Nerve Stimulation-treated Before: $ 4,826 After: $ 2,496	n/a	As a result of offering epilepsy surgery and VNS to the patients, the costs of the most expensive patient group are reduced to the mean cost level of patients with refractory epilepsy. It takes some years to balance all direct costs incurred by epilepsy surgery and VNS by the savings after better seizure control and fewer hospital admissions.
Cockerell et al. 1994 [4]	Newly diagnosed seizures: £611 (first year); £169 per patient per annum (subsequent years)	n/a	Direct cost of £611 per patient per annum which decreased after eight years of follow-up to £169 per patient per annum.
De Zelicourt et al. 2000 [20]	First year: FF 14 305 Second year: FF 3 766	n/a	Cost during first year sensitive to aetiologic categorisation of seizures and other clinical parameters. Cost during second year sensitive to frequency of seizure and treatment with AEDs.
Guerrini et al. 2001 [16]	Mean annual cost: €1,767 Subgroups: Newly diagnosed epilepsy: €1,907 Seizure remission: €844 Frequent non-drug-resistant seizures: €1,112 Drug-resistant seizures: €3,268	n/a	The cost of epilepsy tends to vary significantly depending on the severity and duration of the disease. Hospital services and drugs are the major sources of costs. The setting of health care plays a significant role in the variation of the costs, even for patients in the same category of epilepsy.
Halpern et al. 2011 [34]	n/a	*1:Private; 2:Medicare age <65; 3:Medicare age ≥65; 4:Medicaid, 5:Uninsured* Outpatient visits 1) $266; 2) $56; 3) $414; 4) $10; 5) $397 Hospital stays 1) $344; 2) $5; 3) $258; 4) $2; 5) $1018 Emergency department 1) $124; 2) $16; 3) $38; 4) $33; 5) $860 Prescription medication 1) $809; 2) $2192; 3) $1446; 4) $524; 5) $1597	Uninsured individuals had significantly fewer outpatient visits with neurologists, and greater antiepileptic drug costs than did those with private insurance. Individuals with Medicaid coverage had similar medical resource utilization but lower out-of-pocket costs compared with privately insured individuals.
Kotsopoulos et al. 2003 [17]	GP: €625 UH: €3,393 EC: €4,292	GP: €84 UH: €1,767 EC: €1,164	Patients from GP appeared to have lower direct costs. The cost items anti-epileptic drugs, hospital services, unpaid care, and transportation accounted for the majority of the total direct costs.
Langfitt et al. 2007 [17]	Baseline vs Follow up Persisting seizure group: $2,224 vs $2,982 No surgery group: $1,838 vs $2,567 Surgery, seizure free group: $2,294 vs $1,561	n/a	Costs remain stable over 2 years post-evaluation in patients with temporal lobe epilepsy whose seizures persist, but patients who become seizure free after surgery use substantially less health care than before surgery. Further cost reductions in seizure-free patients can be expected as antiepileptic drugs are successfully eliminated.
Pato Pato et al. 2011 [22]	€2,110 per year (€ 1055 for 6 months)	n/a	See table 3
Tetto et al. 2002 [19]	NDE: €1002; SR: €412; OS: €558; NDR: €1626; DR: €2198; SC: €3945	n/a	The direct costs of epilepsy vary significantly depending on the severity of the disease and the response to treatment. Hospital admissions and drugs are the most common items of expenditure.
Balabanov et al. 2007 [24]	Patients on Carbamazepine Up to 2 adverse events (AEs): €339 2 or more AEs: €806 Patients on Valproate monotherapy Up to 2 AEs: €581 2 or more AEs: €555	n/a	Age, gender and type of seizure did not cause major differences in direct costs. In Carbamazepine patients costs were influenced by the incidence of AEs, time between seizures and percentage of seizure reduction. In Valproate patients costs were influenced by the time period between seizures.
Lagunju et al. 2011 [32]	n/a	Median direct costs for one year AED costs: US$288 In-patient care: US$333 Investigation costs: US$80 Out-patient costs: US$32 Transportation: US$20 Home care: US$800	Carers of children with epilepsy incur very high out-of-pocket expenses due to a lack of well-established national health insurance programme and social support services.
Doumbia-Outtara et al. 2010 [31]	n/a	Mean direct costs of hospitalisation: 148 715 FCFA Examination: 74 FCFA Accommodation: 58 FCFA Anti-epileptic medicines: 17 FCFA	Phenobarbital was the most frequently used AED (40%) and is the treatment of choice for patients. Financial accessibility to modern treatment of epilepsy is difficult as the cost of care is very high compared to the average salary. 22% of patients left the unit prematurely due to lack of financial means.
Dongmo et al. 2003 [30]	n/a	Average cost of treatment per patient: 31 CFA/day	Phenobarbital was the most frequently used AED (75%). Compliance rate was 71% and the main reason for non-compliance was a lack of finances.
Haroon et al. 2012 [29]	n/a	Direct cost to epilepsy patients prescribed 1-4 AEDs 1 AED: Rs5943 2 AEDs: Rs8429 3 AEDs: Rs10091 4 AEDs: Rs10683	The direct cost to patients increased linearly with the addition of AEDs to patients’ prescription. The majority of patients belonged to the lower middle income group. Some newer AEDs had a higher monthly cost (lamotrigine, levetiracetam and lacosamide) compared to older AEDs. Clobazam had the lowest cost of all newer AEDs.
Strzelcyck et al. 2013 [23]	Direct costs per patient (2003 cohort) Anticonvulsant drugs: €600 Hospitalisation: €280 Rehabilitatoin: €90 Diagnostic work-up: €20 Outpatient care: €10 Physical treatment: €10 Special equipment: €3 Total: €1010 Direct costs per patient (2008 cohort) Anticonvulsant drugs: €729 Hospitalisation: €350 Rehabilitatoin: €112 Diagnostic work-up: €25 Outpatient care: €13 Physical treatment: €13 Special equipment: €4 Total: €1266	n/a	Direct costs shifted during the 5-year period of evaluation of trends and resource. During this time hospital costs increased and a cost-neutral increase was observed in the prescription of ‘newer’ AEDs.
Vlasov et al. 2010 [27]	Direct cost of seizures per patient (employed) Primary generalized: 80 124,61 RUB Secondary generalized: 84 006,43 RUB Partial focal: 77 099,28 RUB Complex focal: 7014,04 RUB Polymorphic/undifferentiated: 84 461,56 RUB Direct cost of seizures per patients (unemployed) Primary generalized: 67 754,36 RUB Secondary generalized: 76 528,79 RUB Partial focal: 61 384,87 RUB Complex focal: 66 386,91 RUB Polymorphic/undifferentiated: 85 380,58 RUB	n/a	Although direct costs of treatment increased during the study period, the cost-benefit ratio significantly decreased by 2-3 times in all types of seizures. The study found that rational treatment using ‘new’ AEDs would allow a reduction of the total cost of treatment.

*Abbreviations*: *AED* antiepileptic drugs, *DR* drug-resistant seizures, *EC* epilepsy centre, *GP* general practices, *NDE* newly diagnosed epilepsy, *NDR* frequent non-drug-resistant seizures, *OS* occasional seizures, *SR* seizure remission, *SC* surgical, *UH* university hospital, *na* not colleted or not reported.

**Table 3:** Summary of findings – indirect costs

Study	Employment Status	Productivity loss	Income	Indirect costs summary
Das et al. 2007 [25]				Most reported reason for discontinuation was cost (90%). Discontinued group Average annual cost of treatment: Rs.5500 ($110) Income: Rs.12,800 ($256) Continued group Annual cost of treatment: Rs.4500 ($90) Income: Rs.24,400 ($580)
Farmer et al. 1992 [26]				No difference in work days between people with epilepsy and controls. Not enough details provided in published paper to provide quantitative comparison
Helmstaeder et al. 2000 [33]	(Baseline/Followup) N=161: School (30/12) Employed (82/87) Unemployed (11/18) Incapacitated (21/29) House wife/husband (17/15)			Socioeconomic outcomes was poorer in nonsurgical than in surgical patients
Kotsopoulos et al 2003 [17]	Temporally sick (n) GP: 1; UH: 7; EC: 4 Permanently sick GP: 0; UH: 0; EC: 3 Work on therapeutic basis GP: 0; UH: 0; EC: 2 Unemployed GP: 0; UH: 0; EC: 2 Retired GP: 3; UH: 11; EC: 2 Early retirement GP: 0; UH: 2; EC: 1 Part-time employment GP: 1; UH: 1; EC: 0	Production days lost (days/month) GP: 0 UH: 0 EC: 0.26 Productivity loss (hours/month) GP: 0 UH: 0.30 EC: 0.92		People with epilepsy from the EC reported the highest productivity losses and unemployment rates
Lindsten et al. 2002 [21]	(Control/Patients) 1986-1990 (79/47) Employed (76/41) Unemployed (3/3) Student (0/3) 1991-1993 (73/42) Employed (67/38) Unemployed (4/1) Student (2/3) 1994-1996 (68/40) Employed (60/34) Unemployed (5/3) Student (3/3) 1997 (65/39) Employed (63/33) Unemployed (5/3) Student (0/4)		(Control/Patients) 1986-1990 (82/50) From employment (75/33) Sickness allowance (0/5) Study grant/unemployment benefit (3/6) Disability pension (4/4) Other sources (-/2) 1991-1993 From employment (69/33) Sickness allowance (0/4) Study grant/unemployment benefit (4/3) Disability pension (3/5) Other sources (-/2) 1994-1996 From employment (57/28) Sickness allowance (2/4) Study grant/unemployment benefit (7/5) Disability pension (4/6) Other sources (-/1) 1997 From employment (58/27) Sickness allowance (2/2) Study grant/unemployment benefit (6/7) Disability pension (2/6) Other sources (-/1)	After a newly diagnosed unprovoked epileptic seizure, no negative outcomes regarding employment and education. Income increases unless there is an onset of refractory seizures. Income is lower among patients with epilepsy than controls. This difference can be related to overall morbidity.
Pato Pato et al 2011 [22]		€3,058 per year (€1,529 for 6 months)		Indirect costs due to work productivity losses are substantial and substantially more than direct costs
Balabanov et al. 2007 [24]			Days off work, sick leave days and reduction of salary due to incapacitation were calculated for each patient. These costs were not reported.	Not reported
Strzelczyk et al. 2013 [23]		(2003 cohort) Early retirement: €780 Productivity loss due to part-time work/unemployment: €420 Off-days due to seizures: €410 Total indirect costs: €1610 (2008 cohort) Early retirement: €818 Productivity loss due to part-time work/unemployment: €441 Off-days due to seizures: €430 Total indirect costs: €1689		The amount and distribution of indirect cost components did not change significantly between cohorts.
Lagunju et al. 2011 [32]		Median cost of mother out of work for one year: US$ 1280		Thirty-seven (17.2%) of mothers gave up their jobs to take care of their child with epilepsy. The annual income lost by families due to this ranged from US$480 to US$1280. The overall mean cost of loss of employment across all 215 child participants was US$493.
Lv et al. 2009 [28]	Parents of children with epilepsy: Full-time work: (203/263) Part-time work: (38/263) Don’t work for epilepsy: (22/263) Parents of children without epilepsy: Full-time work: (270/270)		Parents of children with epilepsy: Median household income, Yuan/month: 2800 Mean cost of epilepsy, Yuan/month:4164 Parents of children without epilepsy: Median household income, Yuan/month:3000	Indirect costs of childhood epilepsy have a severe impact on parental quality of life (QoL) and psychological health. Unemployment in particular, can lead to extreme economic hardship.

*Abbreviations*: *EC* epilepsy centre, *GP* general practices, *UH* university hospital

### Direct costs – health system

The annual total cost per patient in Italy was €1302 (€ 0.75 = $US 1) [14]. A cost-of-illness study from Spain estimated that the total direct costs per patient for the care of a person with epilepsy was of €1055 per 6 months and the cost of patients attending consultations for epileptic surgery was €2193 [22]. The direct medical costs in France for the first year after newly diagnosed epilepsy were FF14,305 (FF6 = $US1 in 1998) and FF3,766 during second year [20]. One study from the Netherlands [17] and one study from Italy [16] which focused on children and adolescents compared the direct costs among three different health care settings - general practices, university hospitals and an epilepsy centre. They both found that the costs differ significantly between the different settings of health care, even for patients in the same category of epilepsy.

### Determinants of costs and variation in direct costs

Nine of the eighteen studies that reported direct costs compared different items of expenditures and found that drugs and hospital services were the major sources of costs [14–17, 19, 23, 24, 27, 32]. Three studies from Italy reported that the costs of epilepsy varied significantly depending on the severity of the disease and the response to treatment [14, 16, 19]. Two of them found the annual costs of epilepsy were highest in surgical candidates, followed by patients with drug-resistant epilepsy, active non-drug epilepsy, newly diagnosed epilepsy and epilepsy in remission or with occasional seizures [14, 19] A Russian study showed that yearly costs for different types of seizures significantly varied for employed and unemployed participants [27]. After therapy had been optimized using new antiepileptic drugs, four (primary generalized, secondary generalized, partial focal and complex focal) out of five types of seizures, with the exception of polymorphic or undifferentiated seizures were of lower mean cost per year to unemployed patients [27]. In France, the first year costs after newly diagnosed seizures were highly associated with aetiological categorisation of seizures at inclusion and to other clinical parameters such as the number of seizures, age and pattern of seizures or being treated or not by antiepileptic drugs [20]. The costs during the second year had lower variance and were highly related to frequency of seizures and whether the patients were treated with antiepileptic drugs. The highest costs were incurred during the first year after a newly diagnosed seizure.

Two studies found the health care setting was a significant determinant in the variation of direct costs [16, 17]. For instance in the Netherlands, care provided in a general practice setting cost €52 per person per month, whilst in a university hospital it cost €282 and at a specialised epilepsy centre, €357 [17]. These results reflect the varying degrees of severity of epilepsy between the patients treated across the three sites with patients with severe and complex epilepsy most likely treated in the specialised epilepsy centre.

### Direct costs – out-of-pocket costs

As previously reported, six studies from the Netherlands, US, Nigeria, Cameroon, Cote d’Ivoire and India reported out-of-pocket costs. A study from the US [34] and the previously mentioned Dutch study [17] were the only studies that investigated out-of-pocket expenditures in high income countries. In the Netherlands, individuals cared for in a university hospital had the greatest out-of-pocket costs per month (€147) followed by patients cared for in a specialised epilepsy centre (€97) and then general practice (€7).

In the US, out-of-pocket costs for outpatient visits, hospitals stays, emergency department visits and prescription medications were compared between individuals with epilepsy who were uninsured, had Medicaid coverage, had Medicare (<65 and ≥ 65) or private insurance. The uninsured reported the highest out-of-pocket expenditure US $ 1018 for hospital stays. Uninsured individuals also experienced significantly higher per-visit and total costs for emergency department care compared with patients in all other insurance groups. They also paid the most out-of-pocket for prescription medication [34].

In Nigeria and Cote d’Ivoire patients with epilepsy were found to incur substantial out of pocket burdens – for instance, in Nigeria, 50 % of a cohort of children attending a tertiary centre incurred out of pocket costs of over 20 % annual family income [31, 32]. The high costs of drugs relative to income can also result in non-adherence to medication, as observed in Cote d’Ivoire [31]. However, a program to support access to medications was found to be effective in ensuring that the costs of treatment to patients in rural Cameroon were manageable [30]. A study set in India looked specifically at treatment discontinuation and found that it was associated with high out-of-pocket costs, unemployment and low socioeconomic status.

### Indirect costs – productivity loss

Ten studies from nine countries (Germany, Sweden, Spain, China, India, Netherlands, Nigeria, Ecuador and Bulgaria) estimated the indirect costs related to epilepsy. These studies used a variety of ways to identify indirect costs. Three studies measured the costs in monetary units [22, 23, 32]. A Spanish study provided an average annual cost estimate at €1528 for lost production [22]. This was measured on the basis of lost employment to patients and caregivers. A German study also measured on the basis of lost employment to patients, estimating the total cost of lost production to be €1610 over a 3 month period [23]. Finally, a study in the Netherlands showed that the loss of productivity due to illness and found that loss of productivity was greatest for patients cared in a specialised epilepsy centre (0.26 days per month) and there was no loss of productive days for those cared for in a university hospital and general practice [17].

### Indirect costs – employment status

Five of the studies evaluated the employment circumstances of patients with epilepsy [17, 21, 23, 31, 33] and two studies evaluated the circumstances of parents of children with epilepsy, including work capacity, source of income and incapacity rates [28, 32]. A study from Sweden focused on the incapacity rate and the source of income of patients with epilepsy compared to a control group and found that after a newly diagnosed unprovoked epileptic seizure, no negative impact in terms of employment status. However it found that income was lower in patients with epilepsy than in controls [21].

### Determinants of costs and variation in indirect costs

The Spanish study found that the costs varied significantly between different patients [22] 46 % of the patients had no indirect costs at all whereas 30 % of the patients faced costs of between €3001 and €4000 due to loss of employment.

The Dutch study found that patients from an epilepsy centre reported the highest productivity changes and unemployment rates compared with patients from the university hospital and from the general practices [17].

A German study found that the unemployment rate due to epilepsy was higher for non-surgically treated patients with drug-resistant temporal lobe epilepsy compared to surgically treated patients. Freedom from seizures was found to be a significant determinant for socioeconomic outcomes - 64 % of the surgical patients became seizure-free whereas 23 % of the non-surgically treated group achieved freedom from seizures due to modifications in antiepileptic drug treatment [33].

### Outcome measurement

Seven studies assessed direct costs through questionnaires,[14, 16, 17, 23, 24, 32] five obtained data from medical records [15, 18, 27, 30, 31] and five studies used an ad hoc diary to detail information regarding epilepsy care (laboratory and diagnostic tests, outpatient evaluations, hospital admissions) [16, 17, 19, 23, 24] Three studies used a hospital database to analyse the use of the various health-care services [22, 27, 29]. Two studies obtained data from a survey which conducted in-person interviews using standard case report forms [20, 34] and one from a semi-structured interview [19].

The method of ascertainment was similar amongst the ten studies estimating indirect costs. All of them measured their outcome using self-reported data. Seven studies used a questionnaire [17, 21–23, 26, 28, 32], three used a seizure and cost diary [23–25], and one conducted a semi-structured psychosocial interview [21]. One of the studies also collected information on indirect costs from a social insurance database [21].

### Quality of studies

Overall there was a low risk of bias in the studies reviewed (Table 4). The inclusion criteria were clearly defined for over 90 % of studies. Confounders were only accounted for in 50 % of studies so this may have resulted in an over-estimation of the effects reported in the other studies. For the most part, outcomes were assessed using objective criteria and measured in a reliable way indicating a low risk of detection bias in the studies reviewed. Attrition bias may be an issue as 15 of the studies did not report outcome data for participants lost to follow-up. Finally, while appropriate statistical analyses were used in most studies, it is worth noting that the analyses were generally limited to descriptive and univariate analyses.

**Table 4:** Summary of study quality

	1	2	3	4	5	6
Beghi et al. 2004 [14]	Y	Y	Y	Y	?	Y
De Zelicourt et al. 2000 [20]	Y	Y	Y	Y	?	Y
Helmstaedter et al. 2000 [33]	Y	Y	N	?	?	Y
Lindsten et al. 2002 [21]	Y	Y	N	Y	N	?
Pato Pato et al. 2010 [22]	Y	N	Y	?	Y	Y
Das et al. 2007 [25]	Y	Y	N	Y	?	Y
Tetto et al. 2002 [19]	Y	N	Y	?	?	Y
Kotsopolous et al. 2003 [17]	Y	?	N	?	?	Y
Langfitt et al. 2007 [18]	Y	Y	Y	Y	Y	Y
Halpern et al. 2011 [34]	?	Y	N	N	N	Y
Farmer et al. 1992 [26]	Y	?	N	Y	N	?
Boon et al 2002 [15]	Y	Y	Y	?	Y	?
Guerrini et al. 2001 [16]	Y	Y	?	?	?	?
Cockerell et al 1994 [4]	Y	?	?	N	?	?
Balabanov et al 2008 [24]	Y	Y	N	?	Y	N
Lagunju et al 2011 [32]	Y	Y	Y	N/A	Y	Y
Strzelczyk et al 2013 [23]	Y	Y	Y	?	Y	Y
Lv et al 2009 [28]	Y	Y	?	?	?	?
Doumbia-Outtara et al. 2010 [31]	Y	N/A	Y	?	Y	N/A
Dongmo et al. 2003 [30]	Y	N/A	Y	?	Y	N/A
Haroon et al. 2012 [29]	Y	Y	Y	?	Y	Y
Vlasov et al 2010 [27]	Y	N	?	N	Y	Y

Measurement of study quality was based on the Joanna Briggs Quality Assessment Appraisal checklist available at: http://joannabriggs.org/assets/docs/sumari/ReviewersManual-2011.pdf; 2013

Quality appraisal criteria:

1. Were the criteria for inclusion in the sample clearly defined?

2. Were confounding factors identified and strategies to deal with them stated?

3. Were outcomes assessed using objective criteria?

4. Were the outcomes of people who withdrew described and included in the analysis?

5. Were outcomes measured in a reliable way?

6. Were appropriate statistical analyses used?

## Discussion

Most studies of the economic impact of epilepsy have focused primarily on the direct costs of treatment and have been conducted in high income country settings. In the small number of studies where patients were followed up from initial diagnosis, such costs were found to peak in the initial year of diagnosis, due mainly to surgery or vagus nerve stimulation (VNS). All studies generally reported significant ongoing costs incurred from medications and outpatient medical consultations, with substantially higher costs associated with ongoing seizures.

Out-of-pocket costs were assessed in six studies. One study from the Netherlands assessed out-of-pocket alongside total costs of treatment and found a significantly large component of direct costs (13 % of costs for patients managed by GPs; 52 % managed though a university hospital; 27 % for epilepsy centre) based mainly on the costs of unpaid care and transportation [17]. This finding suggests that much of the direct costs of treatment and ongoing management for patients with epilepsy may slip through the safety net of existing social health insurance schemes. Thus further research in other settings, including those where ostensible universal coverage arrangements are in place, needs to be undertaken to explore the burden of out-of-pocket costs. Such evidence will most likely be specific to health systems and arrangements within them for the reimbursement of health care costs associated with epilepsy.

In Cote d’Ivoire such costs were found to be a major contributor to non-adherence to medications [31]. In such resource-poor settings, where the availability of safety nets in terms of social health insurance and social welfare are limited or non-existent, the potential economic burden on households is likely to manifest in financial catastrophe, under-treatment, poor adherence and treatment abandonment. Other factors beyond costs also come into play, such as the lack of availability of drugs, stigma associated with epilepsy and negative attitudes towards western medicine.

One study that examined the role of private health insurance from the US found that lack of health insurance coverage was associated with fewer visits to neurologists and greater out-of-pockets costs of medicines, compared to those with insurance. Medicaid coverage was found to offer financial protection in terms of lower out-of-pocket payments despite the same health care utilisation as those with private insurance [34].

Where assessed, indirect costs associated with loss of productivity and employment, were shown to have been major sources of burden associated with this condition. These were reflected in reduced employment/productivity, school attendance and income; as very few of these studies provided monetary estimates of these effects, it is not possible to draw conclusions about the relative burden of indirect from direct costs. The conclusions within individual studies were that indirect costs were overwhelming and constituted a significant burden to individuals and societies, and tended to be greater when there were ongoing seizures particularly among those in lower socioeconomic groups. More data are required to confirm the robustness of these findings in other settings.

We recognise that as the majority of the studies relied exclusively on self-reported costs, there is the potential for recall bias to affect the findings. While prospective diaries may mitigate some of the problems associated with self-report by reducing reliance on patient recall, these are often unreliable when patients lack information about the nature of procedures and tests being carried out. Ideally, future research would involve the use of linked administrative data sets for the assessment of direct health care costs. However, where self-reported is likely to be the only feasible means of collecting data on out-of-pocket and indirect costs, the use of prospective diaries and minimising the time between follow-up interviews may improve the reliability of such data.

Nineteen of the twenty-two studies we identified were cohort studies; the others were a case–control and quasi-experimental studies. The length of follow-up in these studies ranged from 3 months to 6 years; most were 12 to 24 months. Whilst the evidence suggests that long-term direct costs of treatment tends to remain steady (at least for those with control over recurrent seizures), data from patients with longer follow-up will provide a better understanding of treatment compliance and its relationship with costs over time.

A major limitation of this review is the poor comparability of findings across studies due to differences in methods and scope. This limited our ability to make direct comparisons of the size of the burden of epilepsy across different populations and health care settings. On the other hand, the merits of such an exercise may be questionable as it may be more relevant to examine the factors associated with variations in cost, such as insurance coverage and category of illness, as a means of providing guidance for policy and the development and targeting of interventions. It was not possible to provide conclusive assessment of risk of bias due to variation in study questions and study designs. Given that the studies included in this review were largely observational and the objective was not to generate an estimate of a pooled treatment effect, such concerns over potential bias can to a large extent be discounted.

A further limitation of this review is that the majority of epilepsy sufferers are found in low and middle income countries in Africa, Central and South America (>80 %) however most of the published research on this topic and so included in this review have been conducted in higher income countries where epilepsy may be a comparably lower burden. It is likely that this review underestimates the true economic burden faced by households in settings where there are fewer resources and a weaker health system capacity to support people with epilepsy and their households. This misalignment between the regions with the greatest disease burden and populations most researched highlights an urgent need for more research in low and middle income countries to guide policy and planning initiatives to address the economic burden of epilepsy in these settings.

## Conclusions

Our review uncovered a small number of varied studies that have examined the costs associated with treatment of epilepsy. The focus of most of the papers was on the direct costs to health systems but a small number also addressed out-of-pocket and indirect costs associated with loss of income and employment. The main findings are that the key drivers of cost are costs of surgery/VNS, and severity and degree of seizure control. In the Netherlands, where patients are ostensibly protected by universal health care programs, significant out-of-pocket costs were evident due to unpaid care and patient transportation. While in the US, health insurance may offset the costs of treatment and enabling access but does not liberate individuals from bearing a significant burden of out-of-pocket costs associated with ongoing costs of managing illness. Given that the magnitude of such costs is inherently context-specific, there is considerable scope for future research in this aspect of epilepsy, particularly in low and middle income countries.

### Additional files

As an appendix, we have provided the search strategy used to obtained the results of this systematic review. As additional files, we have included completed widely accepted systematic review guidelines: the Preferred Reporting Items for Systematic Reviews and Meta-Analyses (PRISMA) and Guidelines for Meta-Analyses and Systematic Reviews of Observational Studies (MOOSE). In addition, we have provided the data extraction form employed by the authors to undertake data extraction for each included paper.

## Appendix

### Search Strategy

Stage 1:

1. exp Epilepsy/
2. epileptic$.mp.
3. seizure$.mp. or exp Seizures/
4. or/1-3
5. DEPRESSION/or DEPRESSION, INVOLUTIONAL/
6. mental disorders/or adjustment disorders/or exp anxiety disorders/or dissociative disorders/ or exp mood disorders/or neurotic disorders/
7. crying/or laughter/or affective symptoms/or exp emotions/or depression/
8. behavioral symptoms/or exp aggression/or motivation/or drive/or behavior/or fatigue/or exp suicide/or anxiety/
9. (tearful$ or apprehens$ or uneas$ or mania or laugh$ or cry$ or suicid$ or dysthymi$ or panic or fear or apathy or anger or aggressi$).mp.
10. nonverbal communication/
11. ((mental or psychiatric or negative or bipolar or dysthymic) adj symptom$).mp.
12. ((mental or affective or bipolar or dysthymic or sleep) adj disorder$).mp.
13. (emotion$ or depress$ or anxiety or fatigue or mood or irritability or worry or tension or distress$ or neuroses or dysthymi$).mp.
14. Cognition Disorders/or cognit$ disord$.mp. [mp = title, abstract, original title, name of substance word, subject heading word, protocol supplementary concept, rare disease supplementary concept, unique identifier]
15. or/5-14
16. exp Employment/
17. employ$.mp.
18. exp Work/
19. vocation$.mp.
20. perform$.mp.
21. occupation$.mp.
22. exp School/
23. academi$.mp.
24. university$.mp.
25. stud$.mp.
26. or/16-25
27. exp economics/
28. exp income/
29. exp health care costs/
30. cost of illness/
31. exp economics medical/
32. exp economics hospital/
33. economics pharmaceutical/
34. expen$.mp.
35. or/27-34
36. social$.mp.
37. friends/or family/or peers/or isolat$/or interperson$/or support/
38. stigma$.mp.
39. discrimin$.mp. or exp discrimination/
40. or/36-39
41. “Quality of life”/
42. “Value of life”/
43. Quality-adjusted life years/
44. (qaly$ or qald$ or qale$).mp.
45. daly$.mp.
46. health status indicators/
47. (satisfact$ or well$ or activ$ or physical$ or energ$ or life$ or role$ or vital$).mp.
48. or/41-47
49. 4 and 15 and 26 and 35 and 40 and 48

Stage 2:

1. exp Epilepsy/
2. epileptic$.mp.
3. seizure$.mp. or exp Seizures/
4. or/1-3
5. exp economics/
6. exp income/
7. exp health care costs/
8. cost of illness/
9. exp economics medical/
10. exp economics hospital/
11. economics pharmaceutical/
12. expen$.mp.
13. or/5-12
14. 4 and 13

## Additional files

Additional file 1:
**Data extraction form.** (DOCX 24 kb) Additional file 2:
**PRISMA Checklist.** (DOC 64 kb) Additional file 3:
**MOOSE Guidelines.** (DOCX 20 kb)

## Abbreviations

NHMRC: National Health and Medical Research Council
ARC: Australian Research Council
ILAE: International League Against Epilepsy
VNS: Vagus nerve stimulation
