# Supplementary material for: The economic impact of epilepsy: a systematic review
Source: BMC Neurol. 2015 Nov 25;15:245. doi: 10.1186/s12883-015-0494-y (PMC4660784; doi:10.1186/s12883-015-0494-y)
Supplement: Additional file 1: — Data extraction form. (DOCX 24 kb) [file 12883_2015_494_MOESM1_ESM.docx]

**Data extraction form**

*Adapted from:

National Collaborating Centre for Methods and Tools (2008). *Quality Assessment Tool for*

*Quantitative Studies Method*. Hamilton, ON: McMaster University. (Updated 13 April, 2010).

Retrieved from <http://www.nccmt.ca/registry/view/eng/15.html>

The Joanna Briggs Institute. *Appendix VII – MAStARI critical appraisal tools*. In: Joanna Briggs Institute Reviewers’ Manual: 2011 edition. Available from <http://joannabriggs.org/assets/docs/sumari/ReviewersManual-2011.pdf>; 2013

| **Name of reviewer** |  |
| --- | --- |
| **ID Number / study number** |  |
| **Citation: Author, Year and title** |  |
| **Publication type:** |  |
| **Date reviewed** |  |
| **Notes** (i.e. f/u on abstracts) |  |

*SECTION 1: Inclusion criteria*

| **Population:**  People with a diagnosis of epilepsy |  |
| --- | --- |
| **Study:**  Observational studies   1. Cohort 2. Case-control |  |
| **Outcome**  Economic outcomes   1. Costs    1. Direct (health care expenses)    2. Indirect (time, productivitiy) 2. Income 3. Others |  |

*SECTION 2: Characteristics of Included Study*

| Author information |  | | |
| --- | --- | --- | --- |
| **Names:**  **Affiliation of the first author and contact details** |  | | |
| **Study information** |  | | |
| **Year of study:** |  | | |
| **Objectives:** |  | | |
| **Country of research** |  | | |
| **Study design** |  | | |
| **Cohort** | 🞏Yes 🞏 No 🞏 Unclear | | |
| **Case-Control Study** | 🞏 Yes 🞏 No 🞏 Unclear | | |
| **Cost-of-Illness Study** | 🞏 Yes 🞏 No 🞏 Unclear | | |
| **Duration (years and months)** |  | | |
| **Length of follow up** |  | | |
| **Participants** |  | | |
| **Recruitment method** |  | | |
| **Total sample size** |  | | |
| **Setting of study** |  | | |
| **Diagnostic criteria of illness/injury** |  | | |
| **Age, mean (SD), range** |  | | |
| **Sex, % male** |  | | |
| **Socio-economic disadvantaged population (i.e. low income, vulnerable group, ethnicity)** |  | | |
| **Co-morbidity** |  | | |
| **Outcomes (repeat rows for each outcome)** | | | |
| **1** |  | | |
| **Outcome name** |  | | |
| **Diagnostic method** |  | | |
| **Name of scale** |  | | |
| **Summary score/index/scores for each domain?** |  | | |
| **Dichotomous or continuous** |  | | |
| **Definition of thresholds** |  | | |
| **Validated measure (Y/N)** |  | | |
| **Validated for use in the population studied? (Y/N)** |  | | |
| **Generic or specific tool** |  | | |
| **Number of domains** |  | | |
| **What concept is underpinning the measure** |  | | |
| **Unit** |  | | |
| **Upper and lower limits**  **Indicate if higher or lower are better** |  | | |
| **Primary, secondary outcomes of the study** |  | | |
| **Timing of assessment** |  | | |
|  |  | | |
| **Results (repeat for each outcome)** |  | |  |
|  |  |  |  |
| ***Continuous variables:*** |  | |  |
| **Costs per patient** |  |  |  |
|  |  |  |  |
|  |  |  |  |

| **Conclusions** |  |
| --- | --- |
| **Key conclusions of the study authors** |  |
| **Policy recommendations of authors** |  |
| **Unintended consequences noted** |  |
| **Misc comments from study authors** |  |
| **References to other studies of interest (that we might have missed)** |  |
| **References to other studies of interest (that might be relevant to the manuscript)** |  |
| **General comments** |  |
| **Comments/concerns of review authors** |  |
