# Supplementary material for: The economic impact of epilepsy: a systematic review
Source: BMC Neurol. 2015 Nov 25;15:245. doi: 10.1186/s12883-015-0494-y (PMC4660784; doi:10.1186/s12883-015-0494-y)
Supplement: Additional file 3: — MOOSE Guidelines. (DOCX 20 kb) [file 12883_2015_494_MOESM3_ESM.docx]

MOOSE Guidelines for Meta-Analyses and Systematic Reviews of Observational Studies*

| **TITLE** | |
| --- | --- |
| Identify the study as a meta-analysis (or systematic review) | **🗹 **  pg 1 |
| **ABSTRACT** | |
| Use the journal’s structured format | **🗹** |
| **INTRODUCTION** presents |  |
| The clinical problem | **🗹**  pg 4 |
| The hypothesis | **🗹**  pg 4 |
| A statement of objectives that includes the study population, the condition of interest, the exposure or intervention, and the outcome(s) considered | **🗹**  pg 4 |
| **SOURCES** describe: | |
| Qualifications of searchers (eg, librarians and investigators) | **🗹**  pg 5 |
| Search strategy, including time period included in the synthesis and keywords | **🗹  **  Appendix |
| Effort to include all available studies, including contact with authors | **🗹**  pg 4-5 |
| Databases and registries searched | **🗹 **  pg 4 |
| Search software used, name and version, including special features used (eg, explosion) | **🗹**  pg 4; Appendix |
| Use of hand searching (eg, reference lists of obtained articles) | **🗹 ** |
| List of citations located and those excluded, including justification | **🗹 **  Available upon request |
| Method of addressing articles published in languages other than English | **🗹**  pg 5 |
| Method of handling abstracts and unpublished studies | **🗹**  pg 6 |
| Description of any contact with authors | **N/A** |
| **STUDY SELECTION** describes: | |
| Types of study designs considered | **🗹**  pg 4-5 |
| Relevance or appropriateness of studies gathered for assessing the hypothesis to be tested | **🗹**  pg 4 |
| Rationale for the selection and coding of data (eg, sound clinical principles or convenience) | **🗹**  pg 5-6 |
| Documentation of how data were classified and coded (eg, multiple raters, blinding, and interrater reliability) | **🗹**  pg 5-6 |
| Assessment of confounding (eg, comparability of cases and controls in studies where appropriate) | **🗹**  Fig 2 |
| Assessment of study quality, including blinding of quality assessors; stratification or regression on possible predictors of study results | **🗹**  pg 11; fig 2 |
| Assessment of heterogeneity | **🗹**  pg 11 |
| Statistical methods (eg, complete description of fixed or random effects models, justification of whether the chosen models account for predictors of study results, dose-response models, or cumulative meta-analysis) in sufficient detail to be replicated | **N/A** |
| **RESULTS** present: | |
| A graph summarizing individual study estimates and the overall estimate | **N/A** |
| A table giving descriptive information for each included study | **🗹 **  Table 1-3 |
| Results of sensitivity testing (eg, subgroup analysis) | **N/A** |
| Indication of statistical uncertainty of findings | **N/A** |
| **DISCUSSION** discuss: | |
| Strengths and weaknesses | **🗹**  pg 14 |
| Potential biases in the review process (eg, publication bias) | **🗹**  pg 13-14 |
| Justification for exclusion (eg, exclusion of non–English-language citations) | **🗹**  pg 5 |
| Assessment of quality of included studies | **🗹**  fig 2 |
| Consideration of alternative explanations for observed results | **🗹**  pg 13-14 |
| Generalization of the conclusions (ie, appropriate for the data presented and within the domain of the literature review) | **🗹**  pg 14 |
| Guidelines for future research | **🗹**  pg 14 |
| Disclosure of funding source | **🗹**  pg 16 |

*Modified from Stroup DF, Berlin JA, Morton SC, Olkin I, Williamson GD, Rennie D, et al. Meta-analysis of observational studies in epidemiology: a proposal for reporting. Meta-analysis Of Observational Studies in Epidemiology (MOOSE) group.

JAMA 2000;283:2008–12. Copyrighted © 2000, American Medical Association. All rights reserved. lists of obtained articles)
